# Investigating the Effects of Dietary Supplementation and High-Intensity Motor Learning on Nutritional Status, Body Composition, and Muscle Strength in Children with Moderate Thinness in Southwest Ethiopia: A Cluster-Randomized Controlled Trial

**DOI:** 10.3390/nu16183118

**Published:** 2024-09-15

**Authors:** Melese Sinaga Teshome, Evi Verbecque, Sarah Mingels, Marita Granitzer, Teklu Gemechu Abessa, Liesbeth Bruckers, Tefera Belachew, Eugene Rameckers

**Affiliations:** 1 Department of Nutrition and Dietetics, Faculty of Public Health, Health Institute, Jimma University, Jimma 378, Ethiopia; teferabelachew2@gmail.com; 2 Rehabilitation Research Centre (REVAL), Rehabilitation Sciences and Physiotherapy, Hasselt University, Wetenschapspark 7, 3590 Diepenbeek, Belgium; evi.verbecque@uhasselt.be (E.V.); sarah.mingels@kuleuven.be (S.M.); marita.granitzer@uhasselt.be (M.G.); teklugem@yahoo.com (T.G.A.); eugene.rameckers@maastrichtuniversity.nl (E.R.); 3 Musculoskeletal Research Unit, Department of Rehabilitation Sciences, Faculty of Kinesiology and Rehabilitation Sciences, Leuven University, 3000 Leuven, Belgium; 4 Department of Special Needs and Inclusive Education, Jimma University, Jimma 378, Ethiopia; 5 I-BioStat, Data Science Institute, Hasselt University, 3590 Hasselt, Belgium; liesbeth.bruckers@uhasselt.be; 6 Research School CAPHRI, Department of Rehabilitation Medicine, Maastricht University, 6200 Maastricht, The Netherlands; 7 Centre of Expertise in Rehabilitation and Audiology, 6281 Hoensbroek, The Netherlands

**Keywords:** moderate acute malnutrition, preschoolers, ready-to-use supplementary food, high-intensity motor learning, muscle strength

## Abstract

Background: In Ethiopia, moderate thinness (MT) is a persistent issue among children. Yet, evidence on the effects of dietary supplementation and motor skills training in these children is limited. Objective: This study aimed to assess the effect of Ready-to-Use Supplementary Food (RUSF), whether or not combined with high-intensity motor learning (HiML), on weight, height, body composition, and muscle strength in children 5–7 years old with MT living in Jimma Town, Ethiopia. Methods: A cluster-randomized controlled trial was carried out among 69 children (aged 5–7) with MT assigned to receive RUSF (n = 23), RUSF + HiML (n = 25), or no intervention (control group, n = 21). A multivariable Generalized Estimating Equations model was used and the level of significance was set at alpha < 0.05. Results:At baseline, there were no significant differences in the outcome measurements between the RUSF, RUSF + HiML, and control groups. However, after 12 weeks of intervention, there were significant mean differences in differences (DIDs) between the RUSF group and the control arm, with DIDs of 1.50 kg for weight (*p* < 0.001), 20.63 newton (N) for elbow flexor (*p* < 0.001), 11.00 N for quadriceps (*p* = 0.023), 18.95 N for gastrocnemius sup flexor of the leg (*p* < 0.001), and 1.03 kg for fat-free mass (*p* = 0.022). Similarly, the mean difference in differences was higher in the RUSF + HiML group by 1.62 kg for weight (*p* < 0.001), 2.80 kg for grip strength (*p* < 0.001), 15.93 for elbow flexor (*p* < 0.001), 16.73 for quadriceps (*p* < 0.001), 9.75 for gastrocnemius sup flexor of the leg (*p* = 0.005), and 2.20 kg for fat-free mass (*p* < 0.001) compared the control arm. Conclusion: RUSF alone was effective, but combining it with HiML had a synergistic effect. Compared to the control group, the RUSF and RUSF + HiML interventions improved the body composition, height, weight, and muscle strength of the studied moderately thin children. The findings of this study suggest the potential that treating moderately thin children with RUSF and combining it with HiML has for reducing the negative effects of malnutrition in Ethiopia. Future research should explore these interventions in a larger community-based study. This trial has been registered at the Pan African Clinical Trials Registry (PACTR) under trial number PACTR202305718679999.

## 1. Introduction

Malnutrition occurs when the body’s nutrient intake does not meet its needs, leading to overnutrition or undernutrition [1]. Undernutrition is measured using anthropometric indicators such as low weight for height, low weight for age, low height for age, and low body mass index (BMI) for age [2,3]. Thinness is a result of acute malnutrition, with severe thinness being defined as a BMI-for-age of <−3 SD and moderate thinness (MT) being defined as a BMI-for-age of ≥−3 to <−2 SD in children aged 5 and older [4]. Body composition, specifically the ratio of fat mass (FM) to fat-free mass (FFM), is an important indicator of undernutrition determining both healthy and ill children’s short-term survival [5].

Worldwide, a total of 45 million children under the age of five (6.8%) were estimated to be wasted in 2022, with 13.7 million (2.1%) being severe cases. Asia accounts for about three-quarters of the children with severe wasting, whereas Africa accounts for 22% of cases [6]. Although the prevalence of childhood undernourishment is decreasing globally, a recent systematic review of studies on low- and middle-income countries (LMICs) found that underweight and thinness remained prevalent among school-age children (6–12 years), with rates ranging from 21% to 36% [7].

Moderate thinness (MT) is more prevalent than severe acute malnutrition (SAM), accounting for about 64% of all cases of acute malnutrition. Undernutrition is responsible for approximately 45% of all child deaths worldwide [8]. In Ethiopia, the prevalence of wasting among children under five ranges from as low as 4% in Addis Ababa to as high as 26% in the Afar Region, and 9% in Oromia [9]. Malnutrition contributes significantly to increased morbidity and mortality throughout an individual’s lifespan [1]. Children who are under-nourished during their school years experience stunted growth, poorer academic achievement, decreased muscle mass, decreased stature, and decreased work capability [8,10]. They also have a high risk of mortality and suffering lifelong detrimental effects on their brain function, behavior, and overall health outcomes if they survive [11,12,13,14,15,16].

Children with moderate acute malnutrition (MAM) are highly vulnerable and need prompt treatment to prevent their condition from deteriorating into SAM. Children with MAM not only have a three-fold higher risk of mortality compared to well-nourished children but they also are more prone to infections and experiencing impaired physical and cognitive development. Timely intervention is imperative to prevent further complications and ensure their healthy growth [17]. Currently, there is a shortage of available nutritional data pertaining to children in middle childhood (aged 5–10 years old) across both regional and international databases [7]. In children, the daily caloric requirement is based on the child’s age, sex, and activity level, with the recommended daily caloric intake increasing as the child grows older. For children aged 4 to 5 years, the recommended intake is 70 kcal/kg/day, and for those aged 6 to 8 years, it is 60 to 65 kcal/kg/day [18]. A study in South Africa has indicated that moderately thin children may have poorer muscle strength, power, and endurance compared to their normal-weight peers and may also experience motor skills difficulties [19]. Evidence shows that children acquire motor skills between the ages of 3 and 8 years old and interventions have been proven to lead to significant improvements in children’s motor skill competence within this particular age range [20,21].

Stodden’s model demonstrates that children who are proficient in fundamental locomotor movement skills (e.g., running, sliding, and jumping) and object control skills (e.g., hitting, catching a ball, and kicking) during childhood are likely to engage successfully in a variety of activities, games, and sports, which positively impact their physical fitness and their future participation in physical activity and weight status [22,23]. One effective strategy to reduce malnutrition in children is through supplementary feeding programs, which provide specialized food products. These products include lipid-based nutrient supplements like Ready-to-Use Supplementary Food (RUSF) and fortified flours such as corn–soy blend (CSB) [24]. Research has shown that RUSFs can improve growth indicators and clinical outcomes in children with mild to moderate malnutrition [25]. RUSF is a lipid-based nutrient spread designed to provide supplemental energy and micronutrients to children with MAM who are also eating other foods. It is energy-dense, has a long shelf life, is resistant to bacterial contamination, does not require any preparation by the end user [26], and is effective in treating MAM [27]. The treatment of children with MAM with lipid-based RUSF stops the progression of MAM to SAM [28]. Recent systematic reviews and meta-analyses indicate that intervention with RUSF is more effective than other dietary interventions in improving the nutritional outcomes and recovery rate of children with MAM [29]. Furthermore, RUSF is very effective in short-term nutritional recovery in children and has shown substantial success among severely and moderately wasted children [30].

One systematic review and meta-analysis has demonstrated that, for children and teenagers with cerebral palsy, a strength training regimen improves muscle strength, balance, gait velocity, or gross motor function without making their muscles more spastic [31]. In a study by Bleyenheuft et al. (2017) [32], goal-oriented training was found to increase motor activity. Comparable research has shown that task-oriented training significantly improves walking and balance in children 4–14 years of age with cerebral palsy [33]. High-intensity motor learning (HiML) is also known for its positive effects, which are achievable within a shorter training time compared to other interventions such as high-intensity interval training [32,34]. To improve general upper limb function, evidence from studies on the unilateral cerebral palsy (CP) population suggests that children need to practice for more than 30–40 h to see improvements in their motor ability [35]. Some studies suggest that 90 h of treatment may be needed to achieve clinically meaningful improvements; however, the exact optimum dosage has not yet been established [36,37,38].

Children enjoy goal-oriented play activities, i.e., active play, because these activities engage them and inspire them to play the same way again. Active play also has numerous other advantages. For instance, research conducted in Indonesia involving children aged 4 to 6 years over 12 weeks found that children who had engaged in active play three times a week had significantly improved motor abilities [39]. In a New Zealand study, active play appeared to be effective in improving cognitive skills in 7–13-year-old children [40]. Another systematic review and meta-analysis showed that task-oriented activities have a positive effect on children with developmental coordination disorder (DCD) [41]. However, there is limited evidence on whether these activities benefit all children, including those with malnutrition. In Ethiopia, the Health Extension Program has been designed to address cases of MAM. However, only standard MAM treatment services are provided in some food-insecure areas, such as in ‘woredas’ under Integrated Management of Acute Malnutrition (IMAM) monitoring [42], and research shows that children with MAM who are not enrolled in supplementary feeding programs experience little improvement and significant deterioration [42].

Currently, there is no evidence on the effects of dietary supplementation (RUSF) and high-intensity motor learning (HiML)—or their combination—on body composition, muscle strength, linear growth, and motor skill competence in 5–7-year-old children with MT. Additionally, MT in this specific age group seems to have been overlooked in rehabilitation research to date. Despite this, at the ages of 5 to 7 years, there is an increased potential to enhance a child’s neuroplasticity [43], which can be realized through improving their muscle strength and motor skills, leading to higher rates of participation. It remains to be explored whether HiML could be more effective when combined with RUSF. This study aims to evaluate the effects of RUSF with and without HiML compared to no intervention on weight, height, body composition, and muscle strength in children with MT aged 5–7 living in Jimma, Southwest Ethiopia.

## 2. Methods

### 2.1. Study Area and Period 

This study was conducted in kindergarten and primary schools of Jimma Town, located 357 km southwest from Addis Ababa, the capital city of Ethiopia. According to data from the Educational Bureau of Jimma Town, there are a total of 27 government kindergarten or zero-grade schools and 25 primary schools in this area. Out of these 27 zero-grade schools, 25 are found within primary schools and the remaining 2 are located separately. There are 3733 students in government kindergarten (zero-grade) schools and 32,443 in primary schools, with 10,123 being in grade one. Students from only three schools—Mendera, Jiren, and Dilfere—were selected for this study. The total number of students was 1325, with 388 being in kindergarten and 937 in primary school. Baseline data were collected from 7 June 2023 to 30 June 2023. Then, an intervention was carried out from 5 July 2023 to 5 October 2023, and endline data were collected from 10 October 2023 to 30 October 2023.

### 2.2. Study Design

A cluster-randomized controlled trial was conducted using a three-arm parallel group design. First, the schools were randomly selected, and then, within these schools, children were randomly selected among the eligible children for the study. The trial adhered to CONSORT guidelines for cluster-randomized trials [44] and was conducted in school settings to promote collective participation. Clusters (schools) were randomized to minimize intervention contamination and for logistical convenience. The three arms included were as follows: arm 1—moderately thin children receiving RUSF; arm 2—moderately thin children receiving RUSF + HiML; and arm 3—the control group (moderately thin children receiving no intervention).

### 2.3. Population and Eligibility

The source population comprised all children aged 5–7 years in zero-grade and grade one in Jimma Town, and the study population comprised randomly selected children with MAM in the zero-grade and grade one. The inclusion criteria were boys and girls 5–7 years old with confirmed MT (with a BMI-for-age z-score of ≥−3 to <−2). The exclusion criteria were as follows: bilateral pitting edema, overt physical disability (such as kyphosis or scoliosis), limb deformities preventing erect standing, visual/auditory problems, neuromotor difficulties, known tuberculosis (TB), HIV/AIDS, suspected allergies to ingredients in the supplements, involvement in an outpatient therapeutic program, skin infection (edematous), any plans to leave the study area within the next 6 months, any other complications, and serious illness.

### 2.4. Sample Size Determination and Sampling Technique

Browne cites a general rule necessitating the use of at least 30 subjects or greater to estimate a parameter when conducting a study [45,46], whereas Julious recommends a minimum sample size of 12 participants per intervention arm as a rule of thumb. Based on the recommendations of Julious (2005) [47] and van Belle (2002) [48] for continuous variables, 12 participants per group is sufficient [46,47]. This is justified based on the rationale of feasibility and precision of the mean and variance [47]. Researchers suggest a sample size of 10–15 in a group to be probably sufficient [49]. Therefore, the optimal sample size for this study was estimated via an ANOVA of the three arms using G*Power version 3.1.9.4, assuming a power of 80%, a precision of 5%, and a medium effect size of 0.5 as there was no previous study, which gave a sample size of 42. Applying a design effect of 1.5 and a loss to follow-up of 10%, the final sample size became 69.

### 2.5. Recruitment of Study Participants and Randomization

School children aged 5–7 years, newly diagnosed with MT based on a BMI-for-age z-score of ≥−3 to <−2, were eligible for participation in this study. Children whose mothers/caregivers consented to participate in the study were enrolled. The clusters (schools) were selected randomly for the intervention and control groups. For each trial arm, one school was randomly selected from the list of schools, and then 25 children who fulfilled the eligibility criteria were randomly selected. The overall sample was divided into the three arms as follows: RUSF, n = 23; RUSF+ HiML, n = 25; and the control group, n = 21. Six of the children refused to participate after they were selected. After enrolment, mothers/caretakers were asked to bring their children to the location of the intervention every day to collect the RUSF rations and receive HiML training. Trained research assistants (n = 8) measured the children’s weight, height, body composition, hand grip strength, and muscle strength at the beginning (baseline data) and the end of the intervention (week 12, endline data). Compliance with the intervention was monitored to ensure that the school children enrolled in the study only consumed the supplementary food supplied. In this study, no children were lost to follow-up. After being assigned to their respective research arms, participants made judgments about whether or not to take the test immediately.

The choice of whether the school was allocated to an intervention group or the control group was blinded. One research team member who had no information about the participants’ identities managed the allocation. The screening procedure was conducted by trained data collectors and facilitated by Health Extension Program workers using anthropometric measurements (BMI-for-age). The children were also checked for bilateral pitting edema. The screening process continued until the sample size was met.

**Figure 1:**
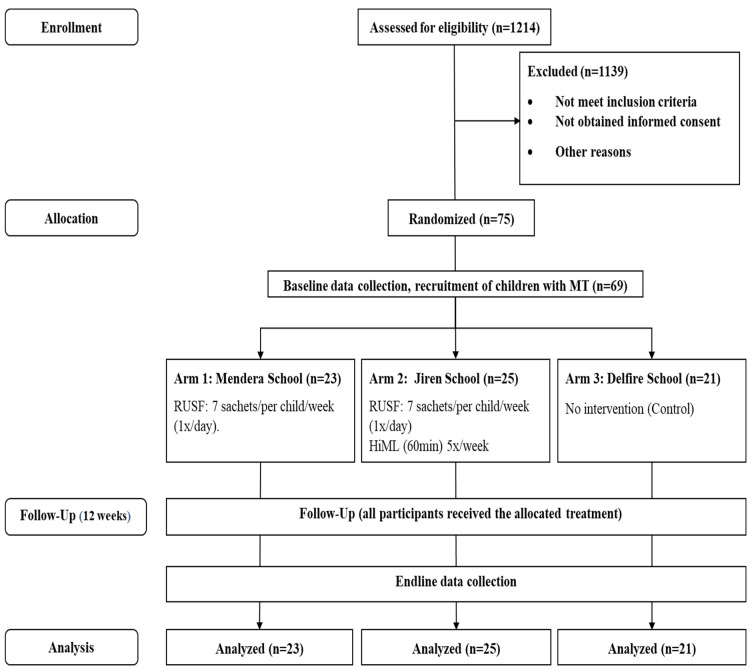
Consort flow diagram, 2023 (n = number of children, MT = moderate thinness, HiML = high-intensity motor learning, RUSF = Ready-to-Use Supplementary Food).

This study used the cluster as the unit of randomization, and the random assignment was conducted by drawing three pieces of paper each labeled with a different cluster number blindly from a bag. The drawn clusters were alternatively assigned to either the intervention groups or the control group (Figure 1).

### 2.6. Interventions

This study was based on providing targeted dietary supplementation (RUSF) and high-intensity motor learning (HiML) for children 5–7 years old who were screened for MT.

#### 2.6.1. Ready-to-Use Supplementary Food (RUSF)

Study participants were allocated to one of the three intervention arms and received the following treatments (Figure 1): arm 1 received 7 sachets of RUSF per child per week (1 sachet per day); arm 2 received 7 sachets of RUSF per child per week (1 sachet per day) and HiML (60 min) five times a week for 12 weeks; and arm 3 received no treatment, acting as the control group. Children included in the study received RUSF in a quantity sufficient to meet their nutrient requirements (500 kcal/day) for 12 weeks, i.e., 7 sachets of RUSF per child per week (1 sachet per day). The required amounts were calculated for 12 weeks (Table A1).

#### 2.6.2. High-Intensity Motor Learning (HiML)

HiML was given five times per week in 60 min sessions. The program or training included fundamental gross motor skills, ball skills, locomotor skills, and cultural or traditional games. The duration of the training regimen was 12 weeks (Table A1). The RUSF rations were distributed daily or weekly over the 12 weeks: weekly for arm 1 and daily for arm 2. The children in arm 2 were served the RUSF in the morning as breakfast after 60 min of HiMl activity, whereas for arm 1, it was given directly in the morning.

### 2.7. Data Collection Methods and Measurement

Data were collected from mothers/caregivers through face-to-face interviews using a structured questionnaire by one physiotherapist, three nurses, and two nutritionists who also supervised the process. The principal investigator and data collectors received prior training that included four days of basic skills training on how to measure isometric muscle strength. The principal investigator then trained the data collectors for one day. Both the family interviews and the children’s measurements were completed at the school during the data collection period. The supervisors performed checks with the principal investigator every day to ensure accuracy. HiML training was provided for 12 weeks by three sports science professionals.

#### 2.7.1. Anthropometric Measurements

Each mother or caregiver was asked for the child’s date of birth, and if they did not remember it, they were asked for an approximate date of birth based on a local events calendar. Measurements were taken according to standard procedures. Each measurement was repeated three times, and the average value was used for analysis. The same person performed all anthropometric measurements to avoid interobserver variation.

Height (cm) was measured using a portable stadiometer (Seca 213, Hamburg, Germany). Children were asked to stand barefoot with their hair pulled back against the wall, looking straight ahead (Frankfurt plane) to ensure that their line of sight was perpendicular to the vertical stand of the stadiometer. Their knees were straight, and their heels, calf, buttocks, and shoulder blades touched the vertical surface of the stadiometer. The resulting height measurement was recorded to the nearest 0.1 cm [50].

Body weight (kg) was measured using a Seca Digital weighing scale (Model 770) made by Seca in Hamburg, Germany. Before each measurement, the scale was calibrated to a zero reading, and the scale was validated daily using a 1 kg standard weight. Each child’s weight was measured while they were barefoot and wearing light clothing. This measurement was recorded to the nearest 0.1 kg [51].

Hand grip strength (kg) was measured using a Takei Digital Grip Strength Dynamometer (model TKK 5401, Tokyo, Japan). The dynamometer was adjusted to fit the participant’s hand size. Participants were asked to hold the dynamometer away from their body using their preferred or dominant hand, with the wrist in a neutral position and the elbow extended. They were then instructed to squeeze the dynamometer with maximal force for 3 to 5 s. Three tests were performed for the preferred or dominant hand, and the average score was recorded.

Muscle strength (Nm) of the upper- and lower-extremity muscles was measured using a digital hand-held dynamometer (HHD), namely the Hoggan MicroFET2™ model, as a manual muscle tester. The muscle strength measurement procedures were preceded by a warm-up for each child. Standard steps were followed to measure the maximum strength of the elbow flexor, knee flexor, and knee extensor muscles [52]. The isometric strength test conducted using the HHD included the following steps: (a) The tester created a fixed and standardized position for the MicroFET 2, and the child tried to move against the MicroFET 2. The sitting position was adjusted to ensure maximum comfort for each child. (b) The maximum force was measured three times. If there was a deviation of more than 20% within these three measurements, a fourth or fifth measurement was performed until the deviation was within the 20% range. (c) Strong verbal encouragement was given during the repetitions so that the children produced maximum force. Three seconds of rest was provided between each measurement. (d) With each attempt, the child gradually built up force against the HHD for about 5 s. (e) When the participant could no longer continue, the measurement was stopped by the participant saying “stop”, and the result was recorded. (f) Three consecutive measurements were performed on the same arm or leg, and the average value was obtained (with a 2 min pause between them). The test procedures are outlined in Table A2.

During the muscle strength test, the child started in a seated position to get used to the process and practice with their leg muscles first, followed by their arm muscles. Testing was then conducted in supine and prone positions. Three attempts were made for each muscle group using the make-test technique, with resistance gradually increasing for about 5 s. A rest time of 30 s was provided between trials, and measurements were alternated between arms and legs to prevent fatigue. To ensure accurate results, the child was encouraged to exert maximum effort in a standardized manner. The highest score for each muscle group was recorded. The hand-held dynamometer (HHD) was placed 5 cm distally from the joint at the segment to allow for a long lever arm and tested at a comfortable location for the child. The testing protocol for children aged 5 to 15 years was adapted from the work of Eek et al. (2006) [53]. The position of the HHD head and the center of the HHD head were marked on the skin, and torque was calculated in newton meters. The testing process took about 15 to 20 min.

#### 2.7.2. Body Composition

A bioelectrical impedance analyzer (BIA) machine, model Bodystat Quadscan 4000 (Bodystat LTD, Isle of Man, UK), was used to assess body composition. Standard procedures were followed for quantifying the FM and FFM of the total body mass [54,55].

### 2.8. Data Quality Control

A trained nurse and nutritionists performed the measurements under standardized conditions. Before taking measurements, all equipment was calibrated and standardized. Calibration was performed by adjusting the indicator to zero before each measurement. The validity of the scales was also checked using an object of known weight. The mothers and/or caregivers of the children were asked for a face-to-face interview.

The questionnaire was prepared in English, then translated into the local language (Afan Oromo), and then translated back into English to check its consistency, and the HiML intervention manual was translated into the local language. The questionnaire was pretested on 5% of the sample children of the same age at another independent school. An intra-class correlation coefficient (ICC) was calculated using the measurements taken at the time of the pretest. The value became 0.93, showing excellent reliability of the muscle strength measurements. The coefficient of variation was 2.1% for height and 1.9% for weight, both of which fall within the acceptable range of less than 3%. Moreover, weekly meetings with the supervisors and data collectors were conducted during the data collection and intervention to address concerns or issues faced by the teams. All of the collected data were checked, cleaned, and coded to avoid any inconsistencies or incompleteness before analysis. Incomplete and inconsistent data were excluded from the analysis.

### 2.9. Statistical Analysis

Data were double-entered into EpiData 3.1 and exported to Statistical Package for Social Sciences (SPSS) version 29 for cleaning and analyses. Descriptive statistics are presented as the mean (±SD) for continuous variables (mainly performance measures) and as proportions for binary variables (mainly sociodemographic variables). A one-way analysis of variance (ANOVA) was performed to compare the performance of children across the three arms of the intervention. Assumptions including the normality of the data were tested using Q-Q plot, histogram, and Shapiro-Wilk tests. The Levene test was used to check the equality of variances. To explore the distribution of sociodemographic variables between the intervention groups, a chi-squared test was performed. The differences between the baseline and endline values were compared based on the intervention status. To account for the differences in certain variables between the intervention and control groups, we employed the difference in differences (DID) approach for analysis. This helped us to compare the effectiveness of the intervention on height, weight, muscle strength, and body composition. By subtracting the baseline measurements from the endline measurements, we generated differences (changes) for all groups, and then compared these differences according to intervention status. The mean DID between the intervention groups was compared using the Multivariable Generalized Estimating Equations model. Statistical significance was interpreted as an alpha value less than 0.05.

## 3. Results

### 3.1. Characteristics of the Parents/Caregivers at Baseline

Table 1 shows an overview of the sociodemographic characteristics of the parents/caregivers. Overall, 61.9%, 47.8%, and 64% of participants in the control, RUSF, and RUSF+ HiML groups were, respectively, mothers or caregivers less than 30 years of age. The family size was less than five members for 90.5% in the control group, 91.3% in the RUSF group, and 52% in the RUSF + HiML group. Of the mothers and caregivers, 85.7% in the control group, 73.8% in the RUSF group, and 80% in the RUSF + HiML group were married. Almost 24% of the mothers were unable to read and write, and 36% had completed primary school in the RUSF + HiML group. Regarding the wealth index, 43.5% of the study participants were poor in the RUSF group and 32% were poor in the RUSF + HiML group (Table 1).

### 3.2. Characteristics of the Children at Baseline

#### Sociodemographic Characteristics

More than half of the children (55.1%) were female. The mean age of the children was 6.33 years (SD: 0.852). The proportion of males (females) was 47.6% (52.4%) in the control group, 47.8% (52.2%) in the RUSF group, and 40% (60%) in the RUSF + HiML group. Five-year-old children represented 26.8% of the control group and 30.4% of the RUSF group. In terms of breastfeeding, 66.7% of the children in the control group and 60% in RUSF + HiML were exclusively breastfed for the first 6 months of their lives. However, 56.5% of children in the RUSF group began consuming complementary foods before they reached 6 months of age. Additionally, 96% of the RUSF + HiML group were fully immunized, and 71.4% of the control group had taken deworming tablets within the last 6 months. Finally, 76.2% of the control group and 76% of the RUSF + HiML group were born in a public health institution (Table 2).

### 3.3. Intervention

At baseline, the mean ± SD values of the RUSF, RUSF + HiML, and no-intervention groups’ arm weight, height, grip strength, elbow flexor, quadriceps, gastrocnemius sup flexor of the leg, fat mass, and fat-free mass were not significantly different.

Table 3 presents a comparison of the height, weight, muscle strength, and body composition data at the baseline and endline of the study. The results show that there was a significant height (*p* < 0.001) and weight (*p* < 0.001) difference. The mean and SD of height was 1.92 ± 1.29 cm (*p* < 0.001), while that of FM was 0.37 ± 1.20 kg (*p* = 0.032) and that of FFM was 1.02 ± 1.85 kg (*p* < 0.001), respectively. Similarly, the difference in differences between the intervention and control groups was 1.64 ± 1.92 (*p* < 0.001) for grip strength, 18.11 ± 13.57 (*p* < 0.001) for elbow flexor, 13.86 ± 14.11 (*p* < 0.001) for quadriceps, and 16.61 ± 13.53 (*p* < 0.001) for gastrocnemius sup flexor of the leg (Table 3).

**Figure 2:**
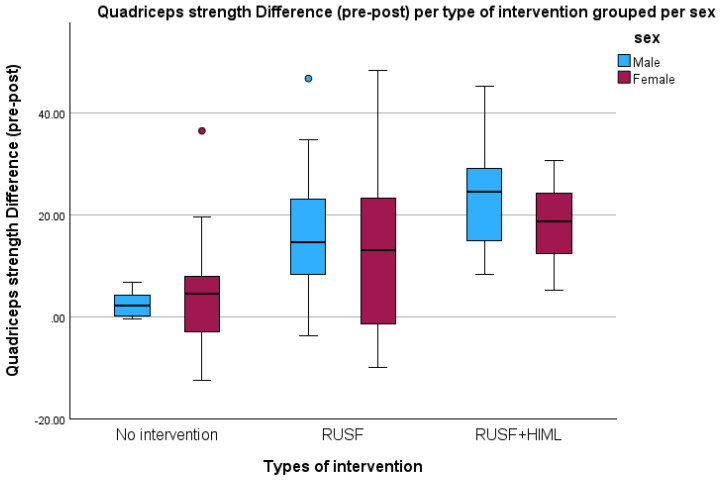
Comparisons of quadricep strength difference per type of intervention, grouped per sex among children aged 5–7 years in Jimma Town, 2023.

**Figure 3:**
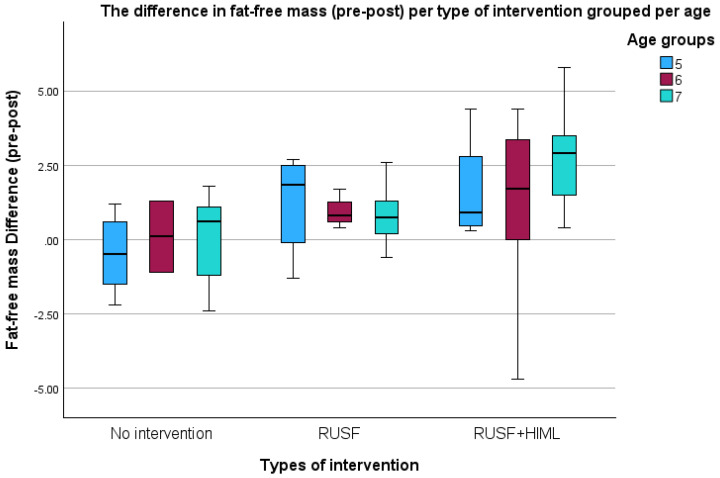
Comparisons of fat-free mass difference per type of intervention grouped per age among children aged 5–7 years in Jimma Town, 2023.

In both outcomes, RUSF + HiML showed a significant improvement over RUSF and the control group (Figure 2 and Figure 3). These figures illustrate the differences in quadricep muscular strength per type of intervention categorized by sex and FFM per age.

After conducting a Post Hoc analysis, differences in the effect of the type of intervention on the dependent variables by their arm of the study were observed. The results showed that the RUSF arm had a significant difference in height, weight, elbow flexor, quadriceps, gastrocnemius sup flexor of the leg, FM, and FFM compared to the no-intervention arm. Similarly, the RUSF + HiML arm had a high difference in height, weight, grip strength, elbow flexor, quadriceps, gastrocnemius sup flexor of the leg, FM, and FFM compared to the no intervention. However, the RUSF + HiML arm had a high difference in height, grip strength, and gastrocnemius sup flexor of the leg compared to the RUSF arm, but no difference was observed in weight, elbow flexor, quadriceps, FM, and FFM (Table 4).

The mean difference in differences was higher in the RUSF arm by 1.20 cm (*p* < 0.001) for height, 1.50 kg for weight (*p* < 0.001), 20.63 for elbow flexor (*p* < 0.001), 11.00 for quadriceps (*p* = 0.023), 18.95 for gastrocnemius sup flexor of the leg (*p* < 0.001), 0.83 kg for fat mass (*p* = 0.021), and 1.03 kg for FFM (*p* = 0.022) compared to no intervention arm. On the other hand, the mean difference in differences was higher in the RUSF + HiML arm by 2.20 cm (*p* < 0.001) for height, 1.62 kg for weight (*p* < 0.001), 2.80 for grip strength (*p* < 0.001), 15.93 for elbow flexor (*p* < 0.001), 16.73 for quadriceps (*p* < 0.001), 9.75 for gastrocnemius sup flexor of the leg (*p* = 0.005), 0.80 kg for FM (*p* = 0.022), and 2.20 kg for fat-free mass (*p* < 0.001) compared to no intervention arm (Table 4).

Participants who were in the RUSF arm showed an increase in their height of 1.21 cm (β = 1.21, *p* < 0.001) compared to those in the no-intervention arm. Similarly, those in the RUSF + HiML intervention group showed an increase in height of 2.28 cm (β = 2.28, *p* < 0.001) compared to the RUSF arm. Regarding weight, participants in the RUSF arm experienced an increase of 1.50 kg (β = 1.50, *p* < 0.001), while those in the RUSF + HiML arm showed an increase of 1.73 kg (β = 1.73, *p* < 0.001) compared to the no-intervention arm.

For grip strength, participants in the RUSF arm showed an increase of 1.04 (β = 1.04, *p* = 0.036), while those in the RUSF + HiML arm demonstrated a larger increase of 2.78 (β = 2.78, *p* < 0.001) compared to the no-intervention arm. Regarding elbow flexor, participants in the RUSF arm experienced an increase of 20.62 (β = 20.62, *p* < 0.001), while those in the RUSF + HiML arm showed an increase of 17.17 (β = 17.17, *p* < 0.001) compared to the no-intervention arm.

Regarding quadricep strength, participants in the RUSF arm showed an increase of 10.29 (β = 10.29, *p* = 0.012), while those in the RUSF + HiML arm demonstrated an increase of 15.89 (β = 15.89, *p* < 0.001) compared to the no-intervention arm. Regarding gastrocnemius sup flexor of the leg, participants in the RUSF arm showed an increase of 19.05 (β = 19.05, *p* < 0.001), while those in the RUSF + HiML arm demonstrated an increase of 9.62 (β = 9.62, *p* = 0.002) compared to the no-intervention arm. Lastly, participants in the RUSF arm showed an increase in fat mass of 1.02 (β = 1.02, *p* = 0.001) and those in the RUSF + HiML arm demonstrated an increase of 0.89 (β = 0.89, *p* = 0.005) compared to the no-intervention arm. On the other hand, participants in the RUSF arm showed an increase in fat-free mass of 0.93 (β = 0.93, *p* = 0.013), while those in the RUSF + HiML arm demonstrated an increase of 2.02 (β = 2.02, *p* < 0.001) compared to the no-intervention arm (Table 5).

## 4. Discussion

In the baseline cross-tab analyses, we found significant differences among the three intervention arms in terms of children who received vitamin A supplementation and complementary feeding before six months, at six months, and after six months. This could be due to the educational status of the mother or caregiver, as well as the socioeconomic status of the family. The results show that after 12 weeks of intervention, moderately thin children who received RUSF or RUSF + HiML demonstrated better improvements in weight, height, FM, FFM, and all strength measures compared to those who received no intervention. However, it was observed that children with MT in the RUSF group gained more elbow flexor muscle and gastrocnemius sup flexor of the leg muscle than those in the RUSF + HiML group. It was also observed that children who received a combination of RUSF and HiML showed significant improvements in height, weight, grip strength, quadricep muscle, and fat-free mass compared to children who received RUSF alone and those in the no-intervention group. Energy is crucial for both catch-up growth and maintenance. Malnourished children suffer from the loss of both lean and fat tissues and require dietary supplementation. The consumption of a food supplement with a high energy density is directly related to increased weight gain, height gain, and muscle strength, as well as improved body composition [56]. Undernutrition is a common condition caused by insufficiencies of nutrients like protein, vitamins, and minerals. This can lead to body composition changes, including decreased muscle mass and body cell mass. As a result, it can cause problems with physical and mental function and lead to poor clinical outcomes. This can make it harder to recover from illness and can increase the risk of death [57]. Our study found that both the children with MT who received RUSF only and those who received RUSF + HiML showed significant improvements in their body composition, muscle strength, and nutritional status. Moreover, a recently conducted systematic review and meta-analysis also found that children with MAM whose diet was supplemented with RUSF showed a significantly improved nutritional status and recovery rate [29].

### 4.1. Impact of RUSF Only

The current study found that RUSF, when implemented for 12 weeks, had a significant positive impact on height, weight, muscle strength, and body composition. This result in relation to RUSF is consistent with the literature [5,58,59,60,61,62]. Supplementation for six to twelve weeks in children under 5 years old [5,25,58,59,61,62,63], but also in children 3–13 years old [60], is beneficial for their FM [58,60,64,65,66], FFM [5,60,62,65,66], BMI [25,60,63], weight [5,25,58,59,61,62,63], and height [25,59,61,63]. However, none of the available studies have established the impact of supplementation on muscle strength, despite the reports suggesting that muscle strength decreases due to undernutrition in under-nourished children relative to their well-nourished peers [24,25,27,29,58,59,60,61,62,63,67]. This result is somewhat surprising, as normally muscle strength is gained when exercise is introduced. For children and adolescents aged 5–17 years, the WHO recommends at least 60 min of moderate- to vigorous-intensity physical activity daily to strengthen their muscles and bones [68,69]. In our study, the children who were part of the group that received only RUSF were expected to show improvements in their anthropometric measurements and body composition, as reported in previous studies [5,58,59,60,61,62], but not in their muscle strength. Notwithstanding, they did gain significant muscle strength in all of the tested muscle groups compared to the group that received no intervention. Perhaps the increase in energy may have increased their overall physical activity and participation throughout the day, but we did not monitor this and therefore cannot know. Future research in this group should investigate the reason for such an increase in muscle strength.

### 4.2. Impact of RUSF Combined with HiML Training

The current study also found that the RUSF + HiML group showed significant improvements in weight, height, body composition, and muscle strength compared to the control group, and the combined group also tended to show more improvements concerning weight gain, height gain, grip strength, quadricep muscle, and fat-free mass. Children learn motor skills sequentially if practicing skills. Fundamental motor skills (FMSs) will be challenged from the ages of 5 to 7 and will change into sports-specific motor skills after a child has reached the age of 7 [70]. There is strong evidence indicating that motor skill interventions have a positive impact on the development of fundamental movement skills (FMSs) in early childhood. Children benefit greatly from these programs, which are usually delivered for 8–12 weeks [71,72]. According to Bardid et al., 2013 [73], a 10-week FMS program had a significant impact on the FMS competency of 3–5-year-old children with motor difficulties. Furthermore, the program helped almost half of these children achieve a normal level of competence. The findings of the present study are new. No previous study has taken on the challenge of combining a dietary supplement with motor skills training because of the underlying fear of challenging the children too much. Our results clearly show that with proper supplementation, a one-hour motor skills training session per day for 12 weeks, focusing on motor learning principles and not fitness principles, is feasible and does not only improve muscle strength but also improves weight, height, and body composition. This means that the children do not have to tap into their nutritional reserves to participate in physical activities. These findings pertaining to the use of interventions with RUSF and combined RUSF + HiML have significant practical implications for the management of MT. This new approach of combining interventions has potential in Ethiopia, where the management of moderately wasted children is only implemented via a few woredas covered by IMAM [42]. Children with MAM in other woredas are left without treatment under the assumption that the Health Extension Program will address them. The findings of this study imply the need for strengthening the implementation of such efforts to reduce the consequences of MT for children.

### 4.3. Implications for Society and Future Research

This study demonstrates that dietary supplementation in the form of RUSF and HiML can be easily implemented in clinical practice to benefit children with MT as well as other populations. Specifically, children with MAM who received RUSF, either alone or combined with functional HiML, showed greater FM and FFM in their body composition, as well as improved linear growth, muscle strength, and overall nutritional status compared to those who received neither intervention.

The current study demonstrates that using RUSF + HiML is feasible. In the future, it is important to explore whether alternative dietary approaches, such as nutrition education, could be equally effective in promoting independence for both mothers and children. This is particularly relevant because RUSF can be expensive and is typically only used in specific cases. Providing education about nutrition and highlighting existing food programs in schools could also be beneficial. Additionally, if children are involved in such programs, they may benefit from increased physical activity.

Insights from the current study suggest that motor training could be effectively integrated into the school curriculum. It is important to train physical education teachers to understand that they can also work with children who are moderately thin by focusing on motor skills rather than solely on cardiorespiratory fitness. This approach could help to involve them in a more holistic societal approach to physical education.

### 4.4. Strengths and Limitations of This Study

Cluster randomization was used to prevent contamination. This approach helped us to control for any confounding factors that may have existed in unmeasured differences across schools. Additionally, all of the factors that differed across schools were included in the initial longitudinal models and retained if they significantly predicted the outcomes (e.g., child age, sex of the child, and monthly income). It is worth noting that the age range of the studied children, from 5 to 7 years, could also be regarded as a limitation as it may not have allowed for much variation in the results and may limit the generalizability of the current results to younger and older children as well. The main limitation of this study is that the children’s dietary intake during intervention was not monitored and the distribution of moderately thin children significantly differed across the age groups of 5, 6, and 7 years, which may have distorted the results.

## 5. Conclusions

The study demonstrates that interventions with RUSF and RUSF + HiML have a significant positive effect on the weight, height, grip strength, elbow flexor, quadriceps, gastrocnemius sup flexor of the leg, fat mass, and fat-free mass of children with MT. The results suggest that such interventions have great potential to curb the emerging burden of malnutrition in Ethiopia, despite this study being conducted in an institutional setting. Future research should examine the sustainability of RUSF and RUSF + HiML through a community-based study with a larger sample size. Further studies are recommended to evaluate the cost-effectiveness of interventions with RUSF and the combination of RUSF + HiML, explore the potential of other dietary approaches such as nutrition education compared to RUSF, and investigate whether HiML can be implemented in school curricula. The results of this study can aid policymakers and stakeholders in making informed decisions and directing resources toward these interventions.

Based on the findings, we conclude that the combination of HiML with RUSF exhibited promising results in enhancing muscle strength and improving body composition in moderately thin children. Therefore, the current study indicates the importance of incorporating this new knowledge into clinical practice for the treatment of children with MT. Given its high potential, more research in necessary in samples of children with MT aged five and older that provide a balanced representation of different age groups.

## Figures and Tables

**Table 1:** Sociodemographic characteristics of parents/caregivers of moderately thin children 5–7 years of age undergoing intervention in Jimma Town, Southwest Ethiopia, 2023 (n = 69).

Variables	Category	Types of Intervention			Total	*p*
		Control (n = 21)	RUSF (n = 23)	RUSF + HIML (n = 25)		
		n (%)	n (%)	n (%)	n (%)	
Marital status of the caregiver	Married	18 (85.7)	18 (78.3)	20 (80.0)	56 (81.2)	0.270
	Divorced	3 (14.3)	1 (4.3)	3 (12)	7 (10.1)	
	Widowed	0 (0.0)	1 (4.3)	1 (4.0)	2 (2.9)	
	Separated	0 (0.0)	0 (0.0)	1 (4.0)	1 (1.4)	
	Single	0 (0.0)	3 (13.0)	0 (0.0)	3 (4.3)	
Age (years) of the mother/caregiver	<30	13 (61.9)	11 (47.8)	16 (64.0)	40 (58.0)	0.478
	≥30	8 (38.1)	12 (52.2)	9 (31.0)	29 (42.0)	
Family size	<5	19 (90.5)	21 (91.3)	13 (52.0)	53 (76.8)	0.001
	≥5	2 (9.5)	2 (8.7)	12 (48.0)	16 (23.2)	
Salary per month (ETB)	<3500	10 (100.0)	19 (100.0)	15 (93.8)	44 (97.8)	0.396
	≥3500	0 (0.0)	0 (0.0)	1 (6.3)	1 (2.2)	
Educational status of the mother	Could not read and write	2 (9.5)	5 (21.7)	6 (24.0)	13 (18.8)	0.956
	Could read and write	2 (9.5)	3 (13.0)	3 (12.0)	8 (11.6)	
	Primary (0–8)	10 (47.6)	8 (34.8)	9 (36.0)	27 (39.1)	
	Secondary (9–12)	5 (23.8)	5 (21.7)	4 (16.0)	14 (20.3)	
	Above secondary (> 12)	2 (9.5)	2 (8.7)	3 (12.0)	7 (10.1)	
Educational status of the husband	Could not read and write	1 (4.8)	2 (8.7)	1 (4.0)	4 (5.8)	0.704
	Could read and write	2 (9.5)	0 (0.0)	1 (4.0)	3 (4.3)	
	Primary (0–8)	7 (33.3)	10 (43.5)	14 (56.0)	31 (44.9)	
	Secondary (9–12)	9 (42.9)	8 (34.8)	6 (24.0)	23 (33.3)	
	Above secondary (> 12)	2 (9.5)	3 (13.0)	3 (12.0)	8 (11.6)	
Occupation of the mother	Housewife	12 (57.1)	7 (30.4)	9 (36.0)	28 (40.6)	0.658
	Merchant	0 (0.0)	1 (4.3)	2 (8.0)	3 (4.3)	
	Gov’t employee	5 (23.8)	6 (26.1)	6 (24.0)	17 (24.6)	
	Self-employee	3 (14.3)	7 (30.4)	5 (20.0)	15 (21.7)	
	Other (e.g., daily laborer, wood seller)	1 (4.8)	2 (8.7)	3 (12.0)	6 (8.7)	
Occupation of the husband	Farmer	0 (0.0)	1 (4.3)	2 (8.3)	3 (4.4)	0.851
	Merchant	1 (4.8)	3 (13.0)	2 (8.3)	6 (8.8)	
	Gov’t employee	8 (38.1)	6 (26.1)	5 (20.8)	19 (27.9)	
	Private employee	10 (47.6)	11 (47.8)	12 (50.0)	33 (48.5)	
	Other (as specified)	2 (9.5)	2 (8.7)	3 (12.5)	7 (10.3)	
Religion	Muslim	9 (42.9)	8 (34.8)	17 (68.0)	34 (49.3)	0.110
	Orthodox	11 (52.4)	11 (47.8)	6 (24.0)	28 (40.6)	
	Protestant	1 (4.8)	4 (17.4)	2 (8.0)	7 (10.1)	
Head of household	Father	15 (71.4)	17 (73.9)	18 (72.0)	50 (72.5)	0.981
	Mother	6 (28.6)	6 (26.1)	7 (28.0)	19 (27.5)	
Wealth index	Poor	3 (14.3)	10 (43.5)	8 (32.0)	21 (30.4)	0.237
	Medium	16 (76.2)	11 (47.8)	13 (52.0)	40 (58.0)	
	Rich	2 (9.5)	2 (8.7)	4 (16.0)	8 (11.6)	
HFIA category	Severe food insecurity	21 (100.0)	23 (100.0)	25 (100.0)	69 (100.0)	
Latrine facility in the compound	Yes	20 (95.2)	21 (91.3)	23 (92.0)	64 (92.8)	0.867
	No	1 (4.8)	2 (8.7)	2 (8.0)	5 (7.2)	
Type of latrine	Flush toilet	0 (0.0)	4 (18.2)	2 (8.7)	6 (9.2)	0.126
	Pit latrine	20 (100.0)	18 (81.8)	21 (91.3)	59 (90.8)	

HFIA: Household Food Insecurity Access Scale. X^2^ analyses. Significant at *p* < 0.05.

**Table 2:** Sociodemographic characteristics of moderately thin children 5–7 years of age in different intervention groups in Jimma Town, Southwest Ethiopia, 2023 (n = 69).

Variables	Category	Types of Intervention			Total	*p*
		Control	RUSF	RUSF + HIML		
		n (%)	n (%)	n (%)		
Sex of child	Male	10 (47.6)	11 (47.8)	10 (40.0)	31 (44.9)	0.825
	Female	11 (52.4)	12 (52.2)	15 (60.0)	38 (55.1)	
Age of child (years)	5	6 (28.6)	7 (30.4)	4 (16.0)	17 (24.6)	0.180
	6	2 (9.5)	2 (8.7)	8 (32.0)	12 (17.4)	
	7	13 (61.9)	14 (60.9)	13 (52.0)	40 (58.0)	
Grade level	KG or Zero-Grade	8 (38.1)	8 (34.8)	12 (48.0)	28 (40.6)	0.623
	Grade One	13 (61.9)	15 (65.2)	13 (52.0)	41 (59.4)	
Place of delivery	At a public health institute	16 (76.2)	19 (82.6)	19 (76.0)	54 (78.3)	0.150
	At a private health institute	1 (4.8)	3 (13.0)	0 (0.0)	4 (5.8)	
	At home	4 (19.0)	1 (4.3)	6 (24.0)	11 (15.9)	
Means of transportation	Walking	21 (100.0)	23 (100.0)	25 (100.0)	69 (100.0)	
Child fully immunized	Yes	19 (90.5)	22 (95.7)	24 (96.0)	65 (94.2)	0.680
	No	2 (9.5)	1 (4.3)	1 (4.0)	4 (5.8)	
Deworming tablet in the last 6 months	Yes	15 (71.4)	18 (78.3)	13 (52.0)	46 (66.7)	0.134
	No	6 (28.6)	5 (21.7)	12 (48.0)	23 (33.3)	
Child receiving vitamin A supplementation	Yes	15 (71.4)	18 (78.3)	6 (24.0)	39 (56.5)	<0.001
	No	6 (28.6)	5 (21.7)	19 (76.0)	30 (43.5)	
Any illness in the past 2 weeks	Yes	11 (52.4)	8 (34.8)	11 (44.0)	30 (43.5)	0.500
	No	10 (47.6)	15 (65.2)	14 (56.0)	39 (56.5)	
EBF for the first 6 months	yes	14 (66.7)	10 (43.5)	15 (60.0)	39 (56.5)	0.273
	No	7 (33.3)	13 (56.5)	10 (40.0)	30 (43.5)	
Complementary feeding	Before 6 months	7 (33.3)	13 (56.5)	10 (40.0)	30 (43.5)	0.030
	At 6 months	1 (4.8)	4 (17.4)	8 (32.0)	13 (18.8)	
	After 6 months	13 (61.9)	6 (26.1)	7 (28.0)	26 (37.7)	
Distance from the school (minutes)	<30 min	20 (95.2)	16 (69.6)	19 (76.0)	55 (79.7)	0.090
	≥30 min	1 (4.8)	7 (30.4)	6 (24.0)	14 (20.3)	
School absenteeism in days	<4 days	20 (95.2)	20 (87.0)	21 (84.0)	61 (88.4)	0.478
	≥4 days	1 (4.8)	3 (13.0)	4 (16.0)	8 (11.6)	

EBF: exclusive breastfeeding. Significant at *p* < 0⋅05.

**Table 3:** Differences in the changes between baseline and endline measurements of height, weight, muscle strength, and body composition among moderately thin children 5–7 years of age in Jimma Town, Southwest Ethiopia, 2023.

Variables	Intervention Type	n	Baseline	Endline	Difference	95% CI		*p*
			Mean ± SD	Mean ± SD	Mean ± SD	Lower Bound	Upper Bound	
Height (cm)	No intervention	21	114.97 ± 7.80	115.69 ± 7.96	0.72 ± 1.11	0.22	1.22	<0.001
	RUSF	23	117.53 ± 7.98	119.45 ± 7.62	1.92 ± 0.70	1.62	2.22	
	RUSF + HiML	25	117.03 ± 5.03	119.95 ± 5.14	2.92 ± 0.96	2.52	3.32	
	Total	69	116.57± 6.97	118.49 ± 7.09	1.92 ± 1.29	1.61	2.23	
Weight (kg)	No intervention	21	17.54 ± 2.40	18.92 ± 2.54	1.38 ± 0.91	0.97	1.80	<0.001
	RUSF	23	17.13 ± 2.27	20.02 ± 2.41	2.89 ± 0.80	2.54	3.23	
	RUSF + HiML	25	17.03 ± 1.73	20.03 ± 1.72	3.00 ± 0.93	2.62	3.39	
	Total	69	17.22 ± 2.12	19.69 ± 2.26	2.47 ± 1.13	2.20	2.74	
Grip strength (kg)	No intervention	21	6.77 ± 2.39	7.05 ± 2.14	0.28 ± 1.04	−0.19	0.75	<0.001
	RUSF	23	7.42 ± 2.24	8.73 ± 2.00	1.32 ± 1.91	0.49	2.14	
	RUSF + HiML	25	5.99 ± 1.69	9.07 ± 1.05	3.08 ± 1.53	2.45	3.71	
	Total	69	6.71 ± 2.16	8.34 ± 1.95	1.64 ± 1.92	1.18	2.10	
Elbow flexor (N)	No intervention	21	68.70 ± 11.88	74.17 ± 12.07	5.46 ± 6.79	2.37	8.55	<0.001
	RUSF	23	69.61 ± 15.03	95.71 ± 19.44	26.09 ± 14.02	20.03	32.15	
	RUSF + HiML	25	71.25 ± 10.14	92.64 ± 11.72	21.39 ± 9.55	17.45	25.33	
	Total	69	69.93 ± 12.33	88.04 ± 17.34	18.11 ± 13.57	14.85	21.37	
Quadriceps (N)	No intervention	21	79.37 ± 17.49	83.51 ± 15.74	4.14 ± 9.69	−0.28	8.55	<0.001
	RUSF	23	79.57 ± 18.08	94.70 ± 19.05	15.13 ± 16.12	8.16	22.11	
	RUSF + HiML	25	76.00 ± 15.20	96.87 ± 13.37	20.87 ± 10.59	16.50	25.24	
	Total	69	78.21 ± 16.73	92.08 ± 16.94	13.86 ± 14.11	10.47	17.25	
Gastrocnemius sup flexor of the leg (N)	No intervention	21	87.12 ± 13.15	93.88 ± 14.35	6.76 ± 8.90	2.71	10.82	<0.001
	RUSF	23	84.91 ± 13.88	110.62 ± 12.88	25.72 ± 14.58	19.41	32.02	
	RUSF + HiML	25	80.14 ± 11.22	96.65 ± 14.30	16.51 ± 9.65	12.53	20.50	
	Total	69	83.85 ± 12.90	100.47 ± 15.49	16.61 ± 13.53	13.36	19.86	
Fat mass (kg)	No intervention	21	5.80 ± 1.22	5.60 ± 1.11	−0.20 ± 0.80	−0.56	0.16	0.032
	RUSF	23	5.98 ± 1.49	6.60 ± 1.31	0.63 ± 1.59	−0.06	1.31	
	RUSF + HiML	25	5.28 ± 1.18	5.88 ± 1.38	0.60 ± 0.92	0.22	0.98	
	Total	69	5.67 ± 1.32	6.03± 1.33	0.37 ± 1.20	0.08	0.65	
Fat-free mass (kg)	No intervention	21	13.40 ± 2.45	13.28± 2.10	−0.12 ± 1.36	−0.74	0.50	<0.001
	RUSF	23	12.57 ± 2.07	13.49 ± 1.78	0.91 ± 1.05	0.46	1.37	
	RUSF + HiML	25	11.97 ± 2.28	14.04 ± 1.50	2.08 ± 2.20	1.17	2.98	
	Total	69	12.60 ± 2.31	13.62 ± 1.79	1.02 ± 1.85	0.58	1.46	

RUSF: Ready-to-Use Supplementary Food; HiML: high-intensity motor learning; SD: standard deviation. Bold values indicate significance (alpha < 0.05). Parameters adjusted for age, sex, family size, and socioeconomic status (wealth index).

**Table 4:** Differences in differences of the effect of the intervention on the dependent variables by their arm of the study (post hoc tests) among moderately thin children 5–7 years of age in Jimma Town, Southwest Ethiopia, 2023.

Dependent Variable	Types of Intervention	Mean Difference	Std. Err	95% CI		*p*
				Lower	Upper	
Height (cm) difference	RUSF vs. No intervention	1.20	0.28	0.64	1.76	<0.001
	RUSF + HiML vs. No intervention	2.20	0.28	1.65	2.75	<0.001
	RUSF + HiML vs. RUSF	1.00	0.27	0.46	1.54	<0.001
Weight (kg) difference	RUSF vs. No intervention	1.50	0.27	0.97	2.03	<0.001
	RUSF + HiML vs. No intervention	1.62	0.26	1.10	2.14	<0.001
	RUSF + HiML vs. RUSF	0.11	0.25	−0.39	0.62	0.653
Grip strength (kg) difference	RUSF vs. No intervention	1.04	0.46	−0.08	2.16	0.074
	RUSF + HiML vs. No intervention	2.80	0.38	1.88	3.72	<0.001
	RUSF + HiML vs. RUSF	1.76	0.50	0.54	2.98	0.003
Elbow flexor (N) difference	RUSF vs. No intervention	20.63	3.28	12.58	28.68	<0.001
	RUSF + HiML vs. No intervention	15.93	2.42	10.06	21.80	<0.001
	RUSF + HiML vs. RUSF	−4.70	3.49	−13.21	3.81	0.379
Quadricep strength (N) difference	RUSF vs. No intervention	11.00	3.97	1.29	20.70	0.023
	RUSF + HiML vs. No intervention	16.73	2.99	9.47	23.99	<0.001
	RUSF + HiML vs. RUSF	5.74	3.97	−3.96	15.43	0.329
Gastrocnemius sup flexor of the leg (N) difference	RUSF vs. No intervention	18.95	3.42	12.11	25.79	<0.001
	RUSF + HiML vs. No intervention	9.75	3.36	3.05	16.46	0.005
	RUSF + HiML vs. RUSF	−9.20	3.28	−15.75	−2.65	0.007
Fat mass (kg) difference	RUSF vs. No intervention	0.83	0.35	0.13	1.52	0.021
	RUSF + HiML vs. No intervention	0.80	0.34	0.12	1.48	0.022
	RUSF+ HiML vs. RUSF	−0.03	0.33	−0.69	0.64	0.938
Fat-free mass (kg) difference	RUSF vs. No intervention	1.03	0.37	0.13	1.93	0.022
	RUSF + HiML vs. No intervention	2.20	0.53	0.90	3.49	<0.001
	RUSF + HiML vs. RUSF	1.16	0.49	−0.04	2.37	0.059

RUSF: Ready-to-Use Supplementary Food; HiML: high-intensity motor learning. Bold values indicate significance (alpha < 0.05).

**Table 5:** Multivariable Generalized Estimating Equations Model results regarding the effect of different interventions on outcome variables among moderately thin children 5–7 years of age in Jimma Town, Southwest Ethiopia, 2023.

Outcome Variable	Predictors		β	95% CI		*p*
				Lower	Upper	
Height (cm)	RUSF + HiML		2.286	1.743	2.830	<0.001
	RUSF		1.206	0.673	1.739	<0.001
	No intervention		Ref.	.	.	.
	Sex (F)		−0.232	−0.648	0.184	0.275
	Age (yrs)	7	−0.057	−0.647	0.533	0.850
		6	0.041	−0.669	0.751	0.910
		5	Ref.			
Weight (kg)	RUSF + HiML		1.730	1.199	2.261	<0.001
	RUSF		1.505	1.030	1.979	<0.001
	No intervention		Ref.			
	Sex (F)		0.417	0.012	0.822	0.044
	Age (yrs)	7	0.244	−0.233	0.722	0.316
		6	0.086	−0.407	0.579	0.733
		5	Ref.			
Grip strength (kg)	RUSF + HiML		2.783	2.017	3.549	<0.001
	RUSF		1.046	0.070	2.022	0.036
	No intervention		Ref.			
	Sex (F)		−0.090	−0.810	0.631	0.807
	Age (yrs)	7	−0.493	−1.318	0.332	0.241
		6	−0.269	−1.667	1.129	0.706
		5	Ref.			
Elbow flexor (N)	RUSF + HiML		17.171	12.359	21.982	<0.001
	RUSF		20.620	14.996	26.244	<0.001
	No intervention		Ref.			
	Sex (F)		−2.400	−7.173	2.373	0.324
	Age (yrs)	7	2.902	−3.049	8.852	0.339
		6	−2.816	−10.057	4.426	0.446
		5	Ref.			
Quadricep strength (N)	RUSF + HiML		15.890	9.303	22.478	<0.001
	RUSF		10.298	2.269	18.328	0.012
	No intervention		Ref.			
	Sex (F)		2.067	−3.964	8.099	0.502
	Age (yrs)	7	−3.619	−10.339	3.100	0.291
		6	−2.975	−11.078	5.128	0.472
		5	Ref.			
Gastrocnemius sup flexor of the leg (N)	RUSF + HiML		9.626	3.613	15.640	0.002
	RUSF		19.055	12.741	25.370	<0.001
	No intervention		Ref.			
	Sex (F)		1.804	−3.265	6.873	0.486
	Age (yrs)	7	4.869	−0.195	9.934	0.060
		6	−5.131	−14.086	3.824	0.261
		5	Ref.			
Fat mass (kg)	RUSF + HiML		0.892	0.275	1.508	0.005
	RUSF		1.020	0.419	1.620	0.001
	No intervention		Ref.			
	Sex (F)		0.047	−0.472	0.565	0.860
	Age (yrs)	7	−0.131	−0.683	0.421	0.642
		6	−0.314	−0.883	0.256	0.280
		5	Ref.			
Fat-free mass (kg)	RUSF + HiML		2.025	0.951	3.100	<0.001
	RUSF		0.932	0.195	1.670	0.013
	No intervention		Ref.			
	Sex (F)		0.609	−0.091	1.309	0.088
	Age (yrs)	7	0.443	−0.373	1.258	0.287
		6	−0.515	−1.895	0.865	0.464
		5	Ref.			

Parameters adjusted for the age and sex of the child, wealth index, and family size of the household. RUSF: Ready-to-Use Supplementary Food; HiML: high-intensity motor learning. Bold values indicate significance (alpha < 0.05).

## Data Availability

The datasets analyzed during the current study are available from the corresponding author upon reasonable request.

## Appendix A

Program

During HiML training, intensity is determined by the amount of hours spent on the task activity, which is undertaken five times per week for 1 h a day over a total duration of 12 weeks, accumulating in 60 h of training overall.

Activity Categories and Subcategories within HiML

A. Gross motor activities. (Subcategory activities: 1. Passing and controlling drills. 2. Shooting drills. 3. Passing, receiving, and dribbling drills. 4. Dribbling and passing from the right side. 5. Dribbling, passing from the left side, and receiving with assistance. 6. Dribbling. 7. Dribbling between four cones, passing, and receiving. 8. Dribbling, passing, and receiving. 9. Dribbling, passing, receiving, and shooting. 10. Dribbling in a zigzag and shooting. 11. Dribbling in a zigzag, passing to a partner, and shooting. 12. Dribbling between two cones, passing, and receiving.)

B. Playground activities. (Subcategory activities: 1. Passing, receiving, and dribbling drills. 2. Shooting into a goal from a triangle placemark. 3. Shooting into a goal from a triangle placemark with assistance. 4. Dribbling and turning around a cone. 5. Dribbling and turning around another player. 6. Dribbling and turning around another player and shooting. 7. Dribbling between four cones, passing, and receiving. 8. Turning, dribbling between four cones, passing, and receiving. 9. Turning, dribbling between two cones, and shooting.)

C. Ball sports activities. (Subcategory activities: 1. One vs. one gameplay. 2. Two vs. two gameplay. 3. Three vs. three gameplay. 4. Two vs. four gameplay. 5. Three vs. five gameplay. 6. Three vs. three playing with the ball. 7. Four vs. four playing with the ball. 8. Four vs. five playing with the ball plus a goalkeeper.)

D. Cultural play activities (e.g., hide and seek, hand dancing). (Subcategory activities: 1. Rosa Rosina. 2. Shumbrarushe-ararushe. 3. Yes-ready. 4. Oringo-papaya.)

E. Cultural activities (e.g., singing, dancing). (Subcategory activities: 1. “Pepsi”. 2. “Chipi-chipi papa”. 3. “Andi Lemendi”. 4. “Dimbish le dimbish”.)

NB:

➢ Three activities are performed every Monday, Tuesday, and Wednesday, and four activities are performed every Thursday and Friday.
➢ The total time spent on activities each day is 1 h (60 min).
➢ The recovery or rest time between each activity is 5 m, and the cooldown time is 5–10 min.
➢ The children rest for 2–3 min between each intensive activity.

**Table A1:** Protocol for the interventions.

Intervention	Dose/Day	Frequency	Duration	Systematic Treatment	Compliance Parameter	Considerations for Implementation	Responsible Person
RUSF	RUSF: 7 sachets/per child/week (1 per day). Follow national guidelines for SFP. 1 packet/day, 100 g/day. 500 kcal; 12.5 g Pro; 31 g Fat; 42.7 g CHO.	Daily follow-up	12 weeks	Guidelines for OTP: If technical capacity and supplies (staff) are available/if health services are available. Deworming; amoxicillin vaccinations; malaria treatment.	Absence of intrahousehold sharing of RUSF rations. Counting of empty sachets.	It can be consumed directly from the package with no dilution, mixing, or cooking necessary.	Research staff; school teachers; Health Extension Program workers; data collectors.
HiML	5 times per week for 60 min. Training of fundamental gross motor skills: Ball skills; Locomotor skills; Cultural games.	Daily	12 weeks		Number of HiML sessions attended by the child.		Research staff; school teachers; parents; Health Extension Program workers; data collectors.

Abbreviations—RUSF: Ready-to-Use Supplementary Food; HiML: high-intensity motor learning.

Isometric strength tests with the HHD:

Tool: Microfet2

Method:

1. Tester creates a fixed position for the Microfet device and the child tries to move against the Microfet2 device.
2. Maximum force is measured 3 times. If a deviation of >20% exists within these three measurements, a fourth or fifth measurement will be performed until the range of 20% is reached.
3. Strong verbal encouragement needs to be given during the repeated measurements so that the child produces maximum force.
4. During each attempt, the child gradually builds up force against the HHD for about 5 s.
5. Positions for placement are standardized. See Table A2.
6. The lever arm is measured between the landmarks with a hard tape measure. See Table A2 below.

**Table A2:** Standard protocol for the hand-held dynamometer measurement of the children’s muscle strength.

Muscle Group	Position	Stabilization	HHD Placement	Direction of Resistance-Creating Block
Elbow flexors (m. biceps brachii)	Sitting/supine. Shoulder adducted, elbow 90° flexed, forearm supinated, and closed fist; or shoulder 30° abducted, elbow 90° flexed, and forearm supinated.	Pelvis stabilized using a belt or manual stabilization.	Pelvis stabilized using a belt or manual stabilization.	Block placed distally to the forearm.
Knee extensor (mm. quadriceps)	Sitting. Hip and knees at 90-degree angles.	Pelvis stabilized in the chair using hands or a belt.	Anterior tibia 5 cm proximal from bimalleolar line.	Block on the frontal side of the tibia.
Knee flexor (mm. hamstrings)	Sitting. Hip and knees at 90-degree angles.	Pelvis stabilized in the chair using hands or a belt.	Anterior tibia 5 cm proximal from bimalleolar line.	Block on the dorsal side of the lower leg.
Ankle plantar flexor (m. gastrocnemius)	Supine. Knees extended, ankle in a neutral position. Foot free from the table.	Pelvis stabilized using a belt or manual stabilization.	Metatarsal heads.	Block on the sole of the foot.
Grip strength	Upright. Elbow bent at a 90° angle.	Handle is adjusted so that the subject’s fingers can grasp and squeeze it.	With Jamar Dynamometer.	Subject squeezes as hard as possible and relaxes.
